# Brugada syndrome: Controversies in Risk stratification and Management

**Published:** 2010-09-05

**Authors:** LM Nunn, J Bhar-Amato, PD Lambiase

**Affiliations:** The Heart Hospital, University College Hospital and Institute of Cardiovascular Sciences, UCL, 16-18 Westmoreland Street, London W1G 8PH

**Keywords:** Brugada syndrome, risk stratification, sudden cardiac death, implantable cardioverter defibrillator, ventricular arrhythmias

## Abstract

In the 18 years since the first description of Brugada Syndrome in a small series of cardiac arrest survivors it has become evident that there is a marked spectrum in phenotype and prognosis. An internal cardiac defibrillator (ICD) is the only established therapy but is associated with significant morbidity. A number of registries have published their data, but risk stratification, particularly in asymptomatic patients, remains controversial. This article summarises the evidence to enable the clinician to make informed management decisions on an individual basis.

## Introduction

Brugada Syndrome (BrS) was first described in 1992 in 8 patients with recurrent episodes of aborted sudden death [1]. When characterising a new disease it is congruent that the most severe phenotype is described first. However, it is now clear that there is a marked spectrum in phenotype reflecting a variable degree of penetrance between individuals from those who are symptomatic with spontaneous type I ECG changes to asymptomatic individuals who only manifest characteristic ECG changes in response to a pharmacological challenge. This creates a challenge in risk stratifying patients and this review will discuss the current controversies in risk stratification and management of this condition.

## Prognosis of Brugada Syndrome

Prior to evaluating individual tools for risk stratifying patients it is important to appreciate the natural history of the disease and event rates. The following should be borne in mind when interpreting registry data and extrapolating this to individual patients.

### Brugada ECG type

**Figure 1:**
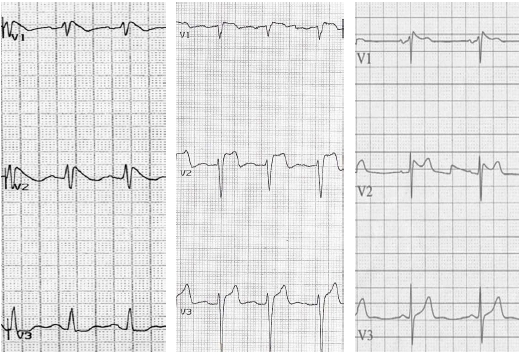
Three types of Brugada ECG pattern recorded in right praecordial leads V1- V3. Type 1 changes are characterised by coved ST-segment elevation of >2 mm (0.2 mV) followed by a negative T wave and is the only ECG phenotype that is currently regarded as diagnostic of Brugada Syndrome 2. Type 2 changes are characterised by saddleback ST segment elevation of >2 mm with a trough of >1 mm ST elevation and a positive or biphasic T wave and may represent a less severe phenotype. Type 3 is characterised by saddleback or coved appearance with an ST-segment elevation of <1 mm.

Three types of Brugada ECG pattern are recognised either spontaneously or following pharmacological provocation (see Figure 1). Currently only a type 1 ECG is regarded as diagnostic of the condition [2]. Of the major published registries two included patients with a non-type 1 ECG. Priori et al [3] included both sets of patients combined in the analysis, with Kamakura et al [4] analysing the two groups separately. It has been argued that including patients with a non-type 1 ECG improves prognosis because these individuals do not have the Brugada syndrome [5]. Whereas others would argue that excluding these patients results in falsely reassuring affected individuals who are still at risk of cardiac arrest [5], albeit a low risk. It has also been established that there can be a significant variation between diagnostic and non-diagnostic ECG pattern in the same patient over time [6]. It is possible that a type 2 ECG represents a milder phenotype of the disease.

### Difficulties in establishing a diagnosis

Establishing a diagnosis can be a challenge. The gold standard has been pharmacological provocation of type 1 ECG changes with a sodium channel blocker in those without spontaneous ECG changes [7]. There is however a discrepancy between the sensitivity of different agents employed [8], the specificity of ECG high lead placement is yet to be established and genotyping is only of limited help with an identifiable mutation present in only 18-30% of cases [2].

### Definition of an arrhythmic event

The arrhythmic end-point in the FINGER registry was defined as an appropriate ICD shock or sudden cardiac death. The authors highlight that with only 7 deaths occurring this was predominantly driven by ICD shocks, and the two are not synonymous [9]. Ventricular tachyarrhythmias can be asymptomatic, terminate spontaneously and would otherwise remain undetected if the patient did not have an ICD. This could lead to an over-estimation of events in those with an ICD, skewing the data for the predictive value of PES as the majority of positive studies will result in ICD implantation compared to a minority of asymptomatic patients with negative PES who receive an ICD [9].

## Risk Stratification

Table 1 summarises the event rates for patients in 3 groups: cardiac arrest survivors; those with a history of syncope; and asymptomatic patients. There has been a clear improvement in prognosis within each group since Brugada et al published their initial registry data. This is most strikingly observed in the asymptomatic group where the cardiac event rate has fallen from 10%/year in 1998 [10] to <1%/year in registries published over the last 5 years [4,9,11]. This most likely reflects the increased diagnosis of patients with a milder phenotype since its first description and referral of less severely affected individuals to international registries. Despite the differences between the groups, all concur that asymptomatic individuals have the best prognosis and those with a history of syncope or prior cardiac arrest have progressively higher risk. A meta-analysis by Gehi et al [12] reported the relative risk (RR) of an event (arrhythmic death, syncope or appropriate ICD shock) was 3.2 in symptomatic patients (history of syncope or aborted sudden cardiac death) compared to asymptomatic patients (p<0.001). RR in patients with a spontaneous rather than sodium channel blocker induced Type I Brugada ECG was higher (RR 4.65) as was male gender (RR 3.5). There was no increase in risk of events for those with a family history of SCD (p=0.97), SCN5a mutation (p=0.18) or inducibility at ventricular stimulation testing (p=0.27). Recently published results from the FINGER registry of 1029 patients (of which 657 had not been previously reported) had similar findings: symptoms and a spontaneous type 1 Brugada ECG were predictors of arrhythmic events, while familial history of SCD, presence of an SCN5A mutation, inducibility of ventricular arrhythmia and in this series gender, was not predictive of arrhythmic events [9]. Family history of sudden cardiac death despite not being predictive for events can still have a role in risk stratification. It is not unreasonable to offer an ICD to asymptomatic type 1 patients who have lost a close relative in this manner and their quality of life has suffered due to anxiety about their personal risk, however small [13]. It is imperative though to ensure such patients are consented appropriately particularly with regard to the morbidity associated with an ICD especially at a young age.

There is clear consensus for secondary prevention. Cardiac arrest survivors are at the highest risk and have a class 1 indication for an ICD [14]. There is a strong evidence base for the prognostic impact of syncope in Brugada syndrome and this is a class 11a indication for ICD [15]. Primary prevention for asymptomatic patients is a more contentious issue. Inducibility of ventricular arrhythmia is greatest amongst symptomatic compared to asymptomatic patients and a third of asymptomatic patients will have inducible ventricular arrhythmia [16] yet inducibility does not seem to equate to risk.

The Brugada registry is the only dataset to show a prognostic impact of inducible ventricular arrhythmia at programmed ventricular stimulation. A meta-analysis of 15 studies comprising 1217 patients conducted by Paul et al [17] did not support the use of ventricular stimulation in risk stratification. Direct comparisons between studies should be interpreted with caution due to heterogeneity amongst the patient populations and stimulation protocols employed. Single and dual site RV stimulation was employed with 2 or 3 extra-stimuli delivered. Some centres employed a particularly aggressive stimulation protocol continuing to deliver ventricular extra-stimuli with a minimal coupling interval of <200msec. With aggressive stimulation VF can be induced in a normal heart [18] questioning the significance of a positive result in these patients under such circumstances.

These differences cannot explain the higher inducibility rates reported in Brugada series as only single site stimulation at the right ventricular apex using minimal coupling interval >200msec was employed [19]. The difference has been ascribed to the inclusion of patients from the early series contributing the majority of events to later series [19]. Combined data presented from Kamakura et al [4] and the FINGER registry [9] shows the risk for arrhythmic events at 4 to 5 years for asymptomatic patients with inducible VF is 2.6% and for asymptomatic patients with spontaneous type 1 Brugada ECG and inducible VF is 2% [20]. This compares to a risk for arrhythmic events of 3.5% for asymptomatic patients who are non-inducible and 3% in asymptomatic patients with spontaneous type 1 ECG Brugada pattern who are non-inducible [20].

### Is a positive ventricular stimulation test useful?

PES is recommended in the 2005 Consensus document for asymptomatic patients with a spontaneous Type I ECG (class IIa) and asymptomatic patients with a drug induced Type I ECG and positive family history of sudden cardiac death (class IIb) [2]. There have been a number of studies published since the consensus document was drawn up all of which have failed to show a prognostic impact from inducibility of ventricular arrhythmia. Univariate analysis of the data from the FINGER registry did show that those with inducible ventricular arrhythmia did have a shorter time to first arrhythmic event than those who were non-inducible (mean event rate of 2.3% per annum compared to 1.2%) [9]. In a study of 220 patients with a type 1 Brugada ECG and ICD implanted there was an 1.5% annual rate of appropriate ICD shock in asymptomatic patients (86% of whom had an ICD implanted following induction of ventricular arrhythmia at testing) [21].

### Is a negative ventricular stimulation test useful?

There is no role for ventricular stimulation testing in symptomatic patients as those who are noninducible still have a significant event rate [22]. Is a negative test in an asymptomatic patient reassuring? Priori reported a 14% false negative rate amongst those who were non-inducible [3] and on meta-analysis of the 23 patients who were asymptomatic and experienced an arrhythmic event, only 61% had inducible ventricular arrhythmia [17]. Analysis of the combined data from recently published Japanese and European registries reports a 3% risk of arrhythmic event at 4-5 years in asymptomatic patients with a type 1 ECG who are non-inducible. Used in the appropriate patient a negative result may be reassuring enough to tip the balance away from ICD implant, but it is imperative that patients should be counselled beforehand what a positive and negative result would mean for them.

Finally, risk stratification is a fluid process and patients should be re-evaluated on a regular basis as phenotype does change with time and new data may materialize.

## Emerging Non-invasive Tools

A variety of non-invasive tools have been assessed which may have a role for risk stratification. Initial results are interesting but need to be borne out in larger cohorts of patients. It is unlikely that any single factor will have 100% sensitivity and specificity, rather the decision to implant an ICD in an asymptomatic patient will be based upon a global assessment of risk.

### Signal Averaged ECG (SAECG)

The presence of late potentials on SAECG has been shown to be more prevalent amongst symptomatic Brugada syndrome patients than asymptomatic patients [23-25], associated with a higher incidence of arrhythmic events [23,24,26,27] and more frequent in patients with inducible VF at programmed ventricular stimulation [28,29].

### Fragmented QRS

Morita et al [30] examined the appearance of multiple small spikes within the QRS complex, termed fragmented-QRS (f-QRS) recorded on 12 lead ECG with a 150Hz low pass filter in 115 patients with Brugada syndrome and spontaneous type 1 changes at rest. F-QRS was identified in 43% of patients, with a higher incidence in the VF group than asymptomatic patients or those with prior syncope (p=0.0069). Over a median of 25 months follow-up those patients with f-QRS and a history of syncope or VF had a higher incidence of recurrent syncope due to VF than those without f-QRS (p<0.001). Interestingly, the presence of late potentials (71% overall) or the inducibility of VF by programmed ventricular stimulation did not predict VF recurrence in this study.

### Infero-lateral early repolarisation

Infero-lateral early repolarisation defined as at least 1mm of J point elevation present as QRS slurring or as a discrete notch inscribed on the S wave in any of the inferior (II, III or aVF) or lateral (I, aVL) leads is present in 11% of BrS patients [31]. They were more likely to be symptomatic at first presentation (p=0.02) and have spontaneous type 1 ECG changes (p=0.05) [31]. A more conventional definition of infero-lateral early repolarisation was employed by Kamakura et al [4] which required the presence of J point elevation in at least 2 of the inferior (II, III or aVF) or lateral (I, aVL and V4-6) leads, resulting in a similar prevalence of 10% of Brugada patients (type 1 and non-type 1). Arrhythmic events occurred more frequently in those patients with infero-lateral early repolarization compared to those without(p<0.005), but there was no difference in risk between the patients with a type 1 ECG and a non-type 1 ECG [4].

## Management of Brugada Syndrome

### General Measures

All patients with Brugada syndrome should receive general advice regarding medications and lifestyle. A number of drugs are contra-indicated in patients with Brugada syndrome as they can provoke the development of Type I changes. A recent list has been published [32] together with a web site for continual updates to provide both physicians and patients with a comprehensive and easily accessible resource. Fever can elicit Type I ECG changes and should be managed aggressively. Patients are advised that sports activities should be limited to those as outlined in the ESC guidelines [33]. Given the dynamic phenotype and prognostic implications of a Type I ECG pattern, patients should have regular out-patient 12 lead ECG recording and 24hr Holter recording.

### Internal Cardiac Defibrillator (ICD)

The role of an ICD in primary and secondary prevention is discussed above. The benefits of an ICD may seem clear; however this has to be balanced against high complication rates, especially in young patients who will require several box changes and have endocardial leads in-situ potentially for many decades. Inappropriate shocks can frequently result from sinus tachycardia or supraventricular arrhythmia (recognised to have a higher incidence in Brugada Syndrome [34]) and this can result in a significant psychological impact especially in young, asymptomatic patients.

ICD outcome studies with a mean or median follow-up of 3.2yrs - 4yrs report appropriate shocks occurring in 8% - 15% of patients [21,35,36]. 0% - 15% of patients implanted for primary prophylaxis [21,35,36], with 22% - 45% of patients implanted following cardiac arrest [21,36]. Overall complications including inappropriate shocks are reported in 20% - 47% of patients [21,35,36]. Inappropriate shocks occur in 20% - 36% of patients caused by sinus tachycardia, supra-ventricular tachycardia, lead dysfunction or dislodgement and T wave over-sensing [21,35,36]. The subcutaneous ICD and absence of an endovascular lead may in the future prove to be an attractive alternative in Brugada syndrome to reduce complications. There is usually no class 1 indication for pacing in this population, but further data regarding the long term risks of a subcutaneous system are awaited.

### Anti-arrhythmic medication

Isoproterenol has been successfully employed to suppress arrhythmic storm in Brugada syndrome [37-39]. A wider role for quinidine is being considered and a registry planned [40]. Quinidine inhibits transient outward K+ current (I_to_), preventing phase II re-entry and ventricular fibrillation in the wedge preparation that mimics Brugada syndrome in vitro [41]. In vivo quinidine attenuates or normalises the Brugada ECG pattern [42,44], render patients non-inducible at PES [42,44-46] and reduces the occurrence of further ventricular arrhythmia [36,42,44,46]. However its usefulness may be tempered by significant side effects, particularly gastro-intestinal disturbance and QTc prolongation.

### Alternatives

Mapping and ablation of ventricular triggers for ventricular fibrillation in Brugada syndrome has been reported [47] and in extreme cases cardiac transplantation is the last option [48].

## Conclusions

There is a clear spectrum in phenotype in the Brugada syndrome. The role of ICD therapy in secondary prevention is established. It is the only effective management in primary prevention but patient selection can be difficult and the majority of patients screened will initially be asymptomatic. The tools for risk stratification are not precise but patient assessment can yield an estimate of risk for arrhythmic events and ICD therapy discussed with them on an individual basis.

## Figures and Tables

**Table 1:**
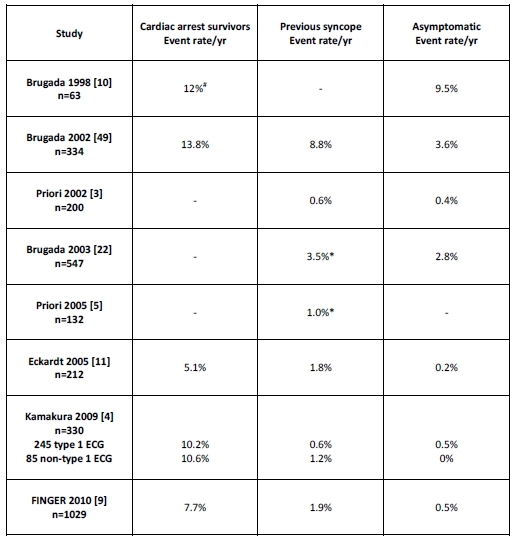
Summary of annual event rates (sudden cardiac death or documented VF) for patients in published registries based upon symptoms

# Combined group of patients: cardiac arrest survivors and those with a history of syncope * Combined group of asymptomatic patients and those with a history of syncope
